# Integration of Transcriptomic Features to Improve Prognosis Prediction of Pediatric Acute Myeloid Leukemia With KMT2A Rearrangement

**DOI:** 10.1097/HS9.0000000000000979

**Published:** 2023-11-22

**Authors:** Jun Li, Suyu Zong, Yang Wan, Min Ruan, Li Zhang, Wenyu Yang, Xiaojuan Chen, Yao Zou, Yumei Chen, Ye Guo, Peng Wu, Yingchi Zhang, Xiaofan Zhu

**Affiliations:** 1 State Key Laboratory of Experimental Hematology, National Clinical Research Center for Blood Diseases, Haihe Laboratory of Cell Ecosystem, Institute of Hematology & Blood Diseases Hospital, Chinese Academy of Medical Sciences & Peking Union Medical College, Tianjin, China; 2 Tianjin Institutes of Health Science, Tianjin, China

## Abstract

Lysine methyltransferase 2A-rearranged acute myeloid leukemia (*KMT2A*-r AML) is a special entity in the 2022 World Health Organization classification of myeloid neoplasms, characterized by high relapse rate and adverse outcomes. Current risk stratification was established on the treatment response and translocation partner of *KMT2A*. To study the transcriptomic feature and refine the current stratification of pediatric *KMT2A*-r AML, we analyzed clinical and RNA sequencing data of 351 patients. By implementing least absolute shrinkage and selection operator algorithm, we identified 7 genes (*KIAA1522*, *SKAP2*, *EGFL7*, *GAB2*, *HEBP1*, *FAM174B*, and *STARD8*) of which the expression levels were strongly associated with outcomes. We then developed a transcriptome-based score, dividing patients into 2 groups with distinct gene expression patterns and prognosis, which was further validated in an independent cohort and outperformed the LSC17 score. We also found cell cycle, oxidative phosphorylation, and metabolism pathways were upregulated in patients with inferior outcomes. By integrating clinical characteristics, we proposed a simple-to-use prognostic scoring system with excellent discriminability, which allowed us to distinguish allogeneic hematopoietic stem cell transplantation candidates more precisely. In conclusion, pediatric *KMT2A*-r AML is heterogenous on transcriptomic level and the newly proposed scoring system combining clinical characteristics and transcriptomic features can be instructive in clinical routines.

## Graphical abstract

**Figure:**
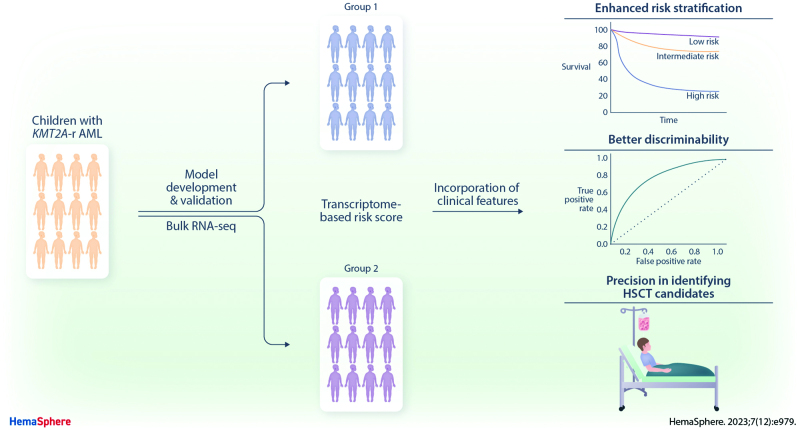

Translocation of 11q23 involving lysine methyltransferase 2A (*KMT2A*, also known as *MLL*) gene is one of the most frequent leukemia-defining abnormalities, which affects both lymphoid and myeloid lineages. It takes up nearly 10% of all pediatric leukemias and is the commonest type of infant leukemia.^1^ Clinically, *KMT2A*-rearranged (*KMT2A*-r) leukemias are characterized by hyperleukocytosis, hepatosplenomegaly, resistance to conventional therapy and with high relapse rate and dismal outcomes. To date, at least 135 fusion partners of *KMT2A* have been described, with *MLLT3*, *MLLT4*, *MLLT10*, and *MLLT1* being the commonest ones in *KMT2A*-r acute myeloid leukemia (AML), and they were also clinically and biologically heterogenous.^2,3^ Despite great advances in molecular biology in recent years, the prognosis of *KMT2A*-r AML did not improve much.

Risk stratification of AML plays a critical role in clinical decision-making. Currently, risk groups were mainly determined by different translocation partners of *KMT2A* gene. In the 2022 ELN guidelines, all adult *KMT2A*-r AML except for *KMT2A::MLLT3* were regarded as high risk with very adverse outcomes.^4^ However, this did not seem to work well in pediatric AML. Yuen et al^5^ demonstrated pediatric AML with *KMT2A::MLLT1* had higher overall survival (OS) and lower relapse rates than *KMT2A::MLLT3*. Balgobind et al^6^ identified AML with *KMT2A::MLLT11* had excellent outcome and failed to confirm favorable outcome of *KMT2A::MLLT3* in pediatric AML. These phenomena indicated greater heterogeneity in pediatric cohorts, suggesting risk stratification solely based on translocation partner is far from adequate. Meanwhile, comparing to other subtypes of AML, traditional predictors such as cytogenetic abnormalities and certain gene mutations provide less informative insights into the prognosis of *KMT2A*-r AML.^7^

In 2013, Groschel et al^8^ identified that high expression level of *EVI1* gene was associated with poor prognosis of *KMT2A*-r AML, which was further validated in patients who underwent allogeneic hematopoietic stem cell transplantation (HSCT).^9,10^ In the meantime, there were studies using RNA-seq data to construct prognostic models for leukemia.^11–13^ These studies suggested transcriptomic feature might serve as a promising biomarker to distinguish patients with different risk levels. Aiming to refine current risk stratification and provide evidence for further research, we analyzed the clinical and RNA sequencing data of pediatric *KMT2A*-r AML and developed a prognostic scoring system that stratified patients into different risk groups with distinct prognosis and biological pathways.

## MATERIALS AND METHODS

### Patients and treatment protocol

Clinical and RNA-seq data of pediatric AML patients were collected from the Therapeutically Applicable Research to Generate Effective Treatments (TARGET) program (available at https://ocg.cancer.gov/programs/target/data-matrix). We included all *KMT2A*-r AML diagnosed between 1997 and 2016. The treatment protocols included AAML1031, AAML0531, AAML03P1, and CCG2961 and were detailed in previous studies.^14–17^ Briefly, all patients received cytarabine-/anthracycline-based regimens. Induction therapies primarily involved daunorubicin or idarubicin, along with cytarabine and etoposide. Individuals who did not achieve complete remission (CR) after 2 induction courses would receive off protocol therapies. Individuals who attained CR and was not allocated to high-risk group, would undergo 2–3 cycles of intensification therapies mainly comprising etoposide and high-dose cytarabine. Patients with high-risk factors would proceed to HSCT after first intensification if a suitable donor was available.

### Definitions of clinical outcomes

CR was defined as bone marrow (BM) blasts <5% by morphology, with no evidence of extramedullary disease (EMD). Relapse was defined as BM blasts ≥5% or presence of EMD after documented CR at the end of course 2. Induction failure was defined as BM blasts ≥5% or evidence of EMD at the end of course 2. Overall survival (OS) was measured from the date of diagnosis to death of any cause or the last date of follow-up. Event-free survival (EFS) was defined as the interval between diagnosis and induction failure, relapse, or death, whichever came the first. Cumulative incidence of relapse (CIR) was defined as the time from diagnosis to relapse for patients who achieved CR at the end of course 2. The competing event for CIR was death without relapse. A 90-day after diagnosis landmark was set, excluding patients who died, relapsed, or lost to follow-up before the landmark.

### Gene expression analysis

Genes that were expressed (RPKM >1) in >30% of samples were further analyzed. The batch effect generated by various library preparation strategies was corrected using the *ComBat* function in the *sva* R package. The differentially expressed genes were identified by the *limma* R package, with an adjusted *P* value of <0.05 and a log2-fold change of >2. The R package *clusterProfiler* was then used to perform gene ontology (GO) and Kyoto Encyclopedia of Genes and Genomes (KEGG) pathway analyses. Over-representation analysis for GO- and KEGG-related terms was assessed with the Fisher’s exact test and corrected for multiple testing by the Benjamini–Hochberg method. Only terms with an adjusted *P* value of <0.01 were considered for GO analyses and *P* value of <0.05 for KEGG analyses.

### Statistical analysis

Continuous variables were presented by median and interquartile range (IQR) and compared by Mann-Whitney *U* test. Categorical variables were presented by percentiles and compared by Fisher’s exact test. Univariable Cox regression was applied to identify the potential genes that are associated with EFS. The least absolute shrinkage and selection operator (LASSO) regression was performed by *glmnet* package in R, to construct a prognostic model based on gene expression levels that were strongly associated with EFS. LASSO regression is a regularization technique that has the effect of shrinking the regression coefficients toward zero, which result in some coefficients being exactly equal to zero. The optimal shrinkage parameter λ, which controls the number of included genes, was determined by 10-fold cross validation of the partial likelihood deviance. Each patient had a risk score calculated according to the model. We used the software X-tile to determine the cutoff value of risk score. X-tile software is a bioinformatics tool that can classify continuous variables into categorical variables based on outcome-based cut-point optimization.^18^ Multivariable Cox regression was applied to investigate the independent prognostic value of factors. The OS and EFS probabilities were estimated using the Kaplan-Meier method and compared by log-rank test using *survival* R package. The CIR was estimated by adjusting for competing risks and was compared by Gray’s test using *tidycmprsk* R package. Model performance was assessed by area under time-dependent receiver operating characteristic (AUROC) curve and compared by the method of Chiang^19^ using *timeROC* R package. All *P* values were 2-sided with a significance level of 0.05. All statistical analyses were performed by R software 4.2.2 (The CRAN project, www.r-project.org).

## RESULTS

### Clinical characteristics of pediatric *KMT2A*-r AML

**Figure 1.**
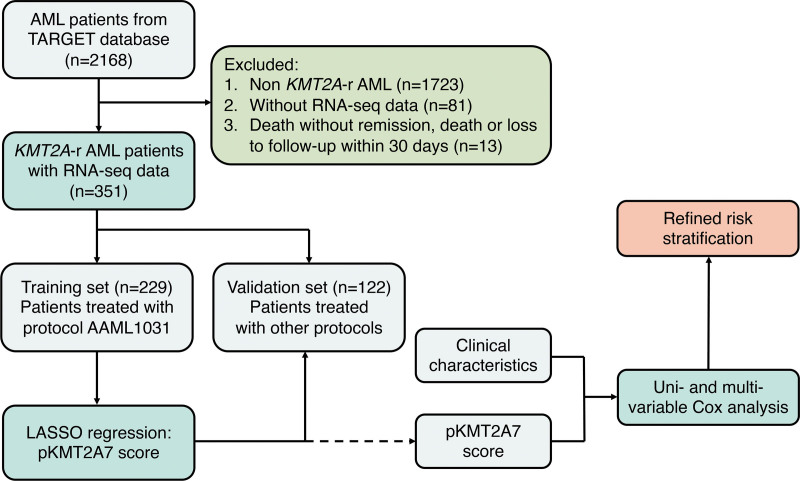
The workflow chart showing the establishmentof the integrated prognosis system.

The flow diagram of this study was shown in Figure 1. A total of 351 pediatric *KMT2A*-r AML patients were included with a median follow-up time of 37.9 (15.6–59.0) months. The patients were divided into 2 independent datasets, training (n = 229) and validation sets (n = 122) according to their treatment protocols. Baseline characteristics were compared and shown in Table 1. There was no statistical significance between OS (53.1% ± 3.5% versus 56.5% ± 4.6%, hazard ratio [HR], 1.11 [95% CI, 0.80-1.55], *P* = 0.531) and EFS (33.2% ± 3.1% versus 33.9% ± 4.3%, HR, 1.06 [95% CI, 0.81-1.39]; *P* = 0.665) of training and validation sets.

**Table 1:** Clinical Characteristics of All Included Patients

Variables	Levels	Overall n = 351	Training set n = 229	Validation set n = 122	*P*
Gender (%)	Female	170 (48.4)	104 (45.4)	66 (54.1)	0.145
	Male	181 (51.6)	125 (54.6)	56 (45.9)	
Age at diagnosis (y, median [IQR])		3.0 [1.0, 12.0]	3.0 [1.0, 12.0]	3.0 [1.0, 13.0]	0.926
WBC at diagnosis (×10^9^/L, median [IQR])		35.6 [8.0, 108.5]	27.7 [6.1, 97.9]	59.4 [16.8, 122.0]	0.001
Bone marrow blast (%, median [IQR])		81.0 [63.3, 90.9]	80.0 [59.8, 89.3]	85.0 [70.3, 92.0]	0.004
Translocation partner (%)	*MLLT3*	114 (32.5)	76 (33.2)	38 (31.1)	0.721
	Other partner genes	237 (67.5)	153 (66.8)	84 (68.9)	
*FLT3*-ITD	No	340 (96.9)	222 (96.9)	118 (96.7)	0.461
	Yes	10 (2.8)	7 (3.1)	3 (2.5)	
	Unknown	1 (0.3)	0 (0.0)	1 (0.8)	
CR status at end of course 1 (%)	CR	271 (77.2)	180 (78.6)	91 (74.6)	0.261
	Not in CR	73 (20.8)	43 (18.8)	30 (24.6)	
	Unevaluable	7 (2.0)	6 (2.6)	1 (0.8)	
HSCT in first CR (%)	No	269 (76.6)	176 (76.9)	93 (76.2)	0.890
	Yes	48 (13.7)	30 (13.1)	18 (14.8)	
	Unknown	34 (9.7)	23 (10.0)	11 (9.0)	

CR = complete remission; FLT3-ITD = FMS-like tyrosine kinase 3-internal tandem duplication; HSCT = hematopoietic stem cell transplant; IQR = interquartile range; WBC = white blood cell count.

### Development of pediatric KMT2A 7 genes (pKMT2A7) score in the training set

Univariable Cox analysis was applied to identify candidate genes of which the expression levels were associated with EFS in the training set. There were 2045 protein coding genes with *P* value of <0.05 in univariable Cox analysis entering LASSO algorithm to construct the prognostic model, which was finalized with 7 genes (*KIAA1522*, *SKAP2*, *EGFL7*, *GAB2*, *HEBP1*, *FAM174B*, and *STARD8*). pKMT2A7 score of each patient was calculated using the coefficients of aforementioned 7 genes in LASSO regression, which was expressed as follows:

pKMT2A7 score = (0.0293 * *KIAA1522* + 0.00934 * *SKAP2* + 0.0000561 * *EGFL7* – 0.00504 * *GAB2* + 0.0142 * *HEBP1* – 0.00319 * *FAM174B* + 0.00519 * *STARD8*)

**Figure 2.**
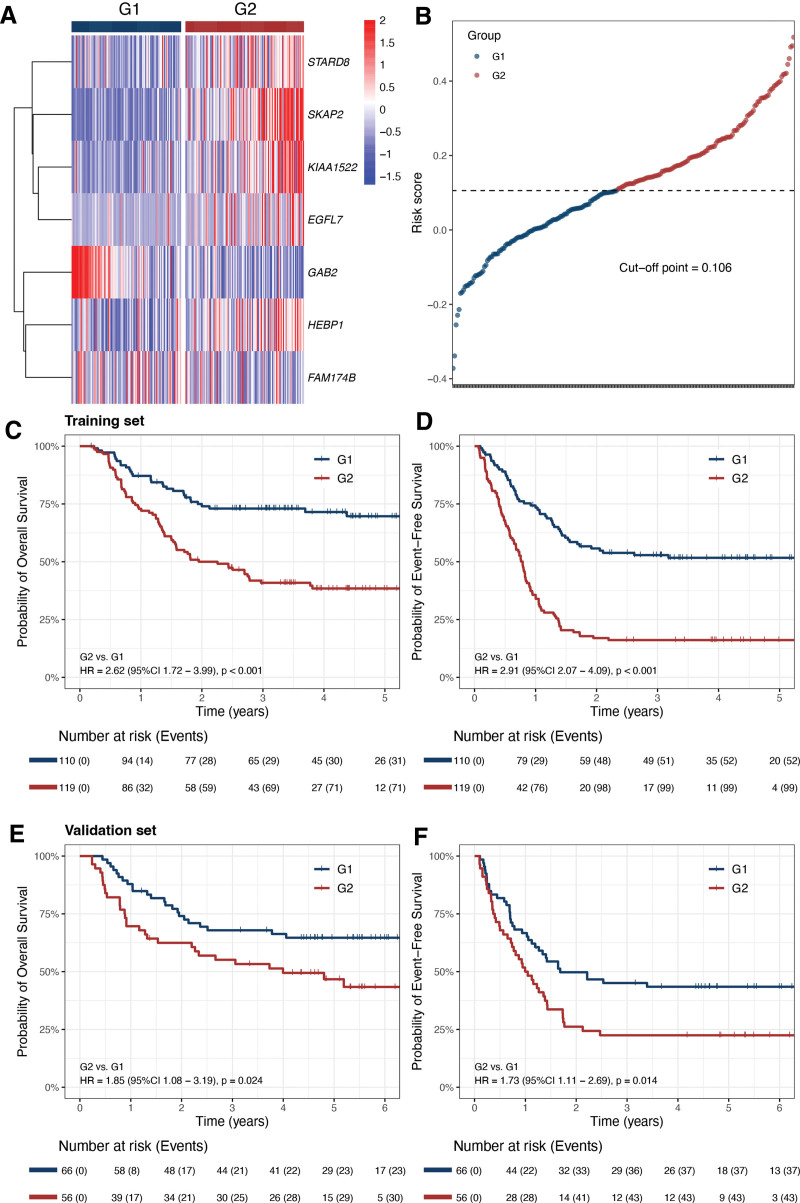
(A) Heatmap of the expression levels of the 7 genes of pKMT2A7 score across patients in training set. (B) The risk score of each patient in the training set. (C,D) Kaplan-Meier curves for OS and EFS rates of G1 and G2 in training set. (E,F) Kaplan-Meier curves for OS and EFS rates of G1 and G2 in validation set. EFS = event-free survival; OS = overall survival.

pKMT2A7 score was a strong predictor for both OS (HR, 1.51 [95% CI, 1.33-1.72], per 0.1 score, *P* < 0.001) and EFS (HR, 1.56 [95% CI, 1.40-1.74], per 0.1 score, *P* < 0.001) as a continuous variable and when divided by quartile (Suppl. Figure S1A,B). The training set was stratified into 2 groups by the cutoff value of 0.106, G1 (n = 110) and G2 (n = 119), with distinct gene expression profiles (Figure 2A,B). The G1 group had pKMT2A7 score <0.106 with higher 5-year OS (69.7% ± 4.7% versus 38.4% ± 4.6%, HR = 2.62 [95% CI, 1.72-3.99], *P* < 0.001) and EFS (51.7% ± 4.8% versus 16.1% ± 3.4%, HR = 2.91 [95% CI, 2.07-4.09], *P* < 0.001) rates than G2 patients (Figure 2C,D). Furthermore, among those with available data for minimal residual disease (MRD) by flow cytometry, G1 had more patients achieving MRD clearance after course 1 (90.7% [98/108] versus 77.6% [90/116], *P* = 0.010) and similar trend was observed for MRD negativity after course 2 (97.7% [86/88] versus 92.2% [83/90], *P* = 0.169). With respect to clinical features, G1 group was characterized by higher proportion of patients under 10 years old (79.1% versus 64.7%; *P* = 0.019) and *KMT2A::MLLT3* fusion gene (40.0% versus 26.9%; *P* = 0.049, Suppl. Table S1).

### The pKMT2A7 score predicts outcome of patients in external validation set

To validate the prognostic value of pKMT2A7 score in an external cohort, the patients in the validation set were also separated into G1 (n = 66) and G2 (n = 56) groups. G1 group had better outcomes than G2 group with higher 5-year OS (64.6% ± 6.0% versus 46.7% ± 6.9%, HR, 1.85 [95% CI, 1.08-3.19], *P* = 0.024) and EFS (43.5% ± 6.2% versus 22.4% ± 5.7%, HR, 1.73 [95% CI, 1.11-2.69], *P* = 0.014) rates (Figure 2E,F). Similarly, G1 group of validation set has more patients under 10 years old (74.2% versus 57.1%; *P* = 0.056) and with *KMT2A::MLLT3* fusion gene (34.8% versus 26.8%; *P* = 0.433), however, without statistical significance due to limited sample size. The MRD data were not available for validation set.

### Identification of distinct gene expression patterns and related biological pathways

**Figure 3.**
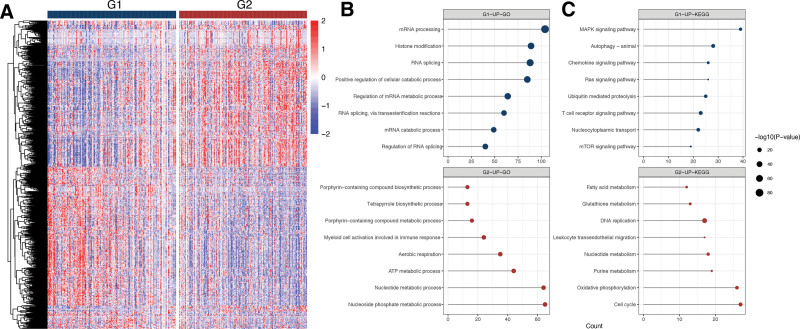
(A) Heatmap of the expression levels of differentially expressed genes between G1 and G2. (B,C) GO enrichment and KEGG pathways analysis of upregulated genes of G1 and G2. ATP = adenosine triphosphate; GO = gene ontology; KEGG = Kyoto Encyclopedia of Genes and Genomes; MAPK = mitogen-activated protein kinase.

G1 and G2 had exhibited disparate gene expression patterns (Figure 3A). To investigate the biological differences, we analyzed the differentially expressed genes of the two groups. A total of 1127 upregulated and 1219 downregulated genes were identified. Then, we implemented GO and KEGG pathway enrichment analyses. According to the findings, the top enriched GO terms for upregulated genes in G2 were mostly related to metabolic process and aerobic respiration and the most significant pathways included cell cycle, oxidative phosphorylation (OXPHOS), and metabolism. As for G1 group, the top enriched GO terms for upregulated genes were mainly associated with mRNA processing, while the top pathways included MAPK, Ras, chemokine and mTOR signaling pathways (Figure 3B,C).

### Performance comparison between pKMT2A7 score and LSC17 score

**Figure 4.**
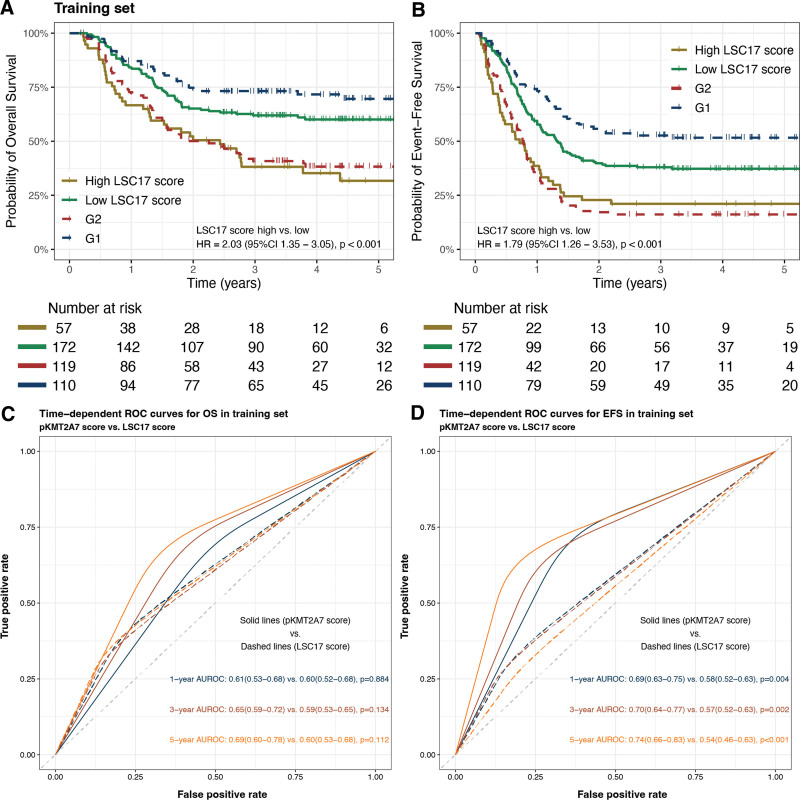
(A,B) Kaplan-Meier curves for OS and EFS rates of high and low LSC17 score groups (solid lines) and pKMT2A7 groups (dashed lines) in training set. (C,D) Time-dependent ROC curves for OS and EFS comparing pKMT2A7 score and LSC17 score in training set. EFS = event-free survival; OS = overall survival; ROC = receiver operating characteristic.

We further compared the performance of pKMT2A7 score and former published LSC17 score.^20^ Because LSC17 score was not originally validated in pediatric cohorts, we used the cutoff value determined by Duployez et al,^21^ which classified LSC17 score greater than the third quartile as high and less than the third quartile as low. We found LSC17 score was also a prognostic factor for both OS (high versus low: 31.7% ± 7.0% versus 60.1% ± 3.9%, HR, 2.03 [95% CI, 1.35-3.05], *P* < 0.001) and EFS (high versus low: 21.1% ± 5.4% versus 37.3% ± 3.7%, HR, 1.79 [95% CI, 1.26-3.53], *P* < 0.001) in the training set (Figure 4A,B). To provide a more intuitive comparison between the two prognostic scores, we evaluated their discrimination abilities by AUROC. The pKMT2A7 score had significantly higher 1-year (OS: 0.61 [95% CI, 0.53-0.74] versus 0.60 [95% CI, 0.52-0.68], *P* = 0.884; EFS: 0.69 [95% CI, 0.63-0.75] versus 0.58 [95% CI, 0.52-0.63], *P* = 0.004), 3-year (OS: 0.65 [95% CI, 0.59-0.72] versus 0.59 [95% CI, 0.53-0.65], *P* = 0.134; EFS: 0.70 [95% CI, 0.64-0.77] versus 0.57 [95% CI, 0.52-0.63], *P* = 0.002), and 5-year (OS: 0.69 [95% CI, 0.60-0.78] versus 0.60 [95% CI, 0.53-0.68], *P* = 0.112; EFS: 0.74 [95% CI, 0.66-0.83] versus 0.54 [95% CI, 0.46-0.63], *P* < 0.001) AUROCs in predicting OS and EFS than LSC17 score in training set (Figure 4C,D). Although in validation set, high LSC17 score also predicted inferior outcomes but did not reach statistical significance (OS high versus low: 48.9% ± 9.3% versus 57.0% ± 5.4%, HR [95% CI], 1.33 [0.73-2.42], *P* = 0.348; EFS high versus low: 21.5% ± 7.7% versus 37.7% ± 5.1%, HR [95% CI], 1.41 [0.87-2.30], *P* = 0.164; Suppl. Figure S2A,B). Similarly, the pKMT2A7 score had higher AUROCs than LSC17 score in validation set as well (Suppl. Figure S2C,D).

### Establishment of a new prognostic system incorporating pKMT2A7 score and other clinical characteristics

To investigate the independent prognostic value of the pKMT2A7 score, we included it into uni- and multivariable Cox analyses along with other potential prognostic factors. Based on prior studies, we chose 10 years old as cutoff value for age at diagnosis.^22,23^ It showed that high pKMT2A7 score was an independent risk factor for both OS and EFS (HR_OS_, 2.10 [95% CI, 1.43-3.11], *P* < 0.001; HR_EFS_, 2.14 [95% CI, 1.55-2.94], *P* < 0.001) together with age at diagnosis of ≥10 years old (HR_OS_, 1.65 [95% CI, 1.11-2.45], *P* = 0.001; HR_EFS_, 1.72 [95% CI, 1.22-2.43], *P* = 0.002) and translocation partner other than *MLLT3* (HR_OS_, 1.67 [95% CI, 1.08-2.60], *P* = 0.022; HR_EFS_, 1.45 [95% CI, 1.04-2.04], *P* = 0.033; Table 2). We further conducted subgroup analyses, and the pKMT2A7 score remained a strong predictor in all subgroups including 10 years older or younger, *MLLT3* and other fusion partners (Suppl. Figure S3A,D).

**Table 2:** Uni- and multivariable Cox analysis of variables impacting OS and EFS

		OS				EFS			
Variables	Groups	Univariable analysis		Multivariable analysis		Univariable analysis		Multivariable analysis	
		HR (95% CI)	*P*	HR (95% CI)	*P*	HR (95% CI)	*P*	HR (95% CI)	*P*
Gender	Male vs Female	1.15 (0.84-1.57)	0.399	0.87 (0.61-1.26)	0.460	1.21 (0.93-1.57)	0.153	0.93 (0.69-1.26)	0.639
Age at diagnosis (y)	<10 vs ≥10	1.93 (1.40-2.66)	<0.001	1.65 (1.11-2.45)	0.014	1.56 (1.19-2.04)	0.001	1.72 (1.22-2.43)	0.002
WBC at diagnosis (×10^9^/L)	<50 vs ≥50	1.23 (0.90-1.69)	0.195	1.30 (0.90-1.90)	0.167	1.22 (0.94-1.58)	0.131	1.08 (0.80-1.47)	0.620
Bone marrow blast (%)	<80 vs ≥80	0.91 (0.65-1.26)	0.568	0.84 (0.58-1.24)	0.385	0.96 (0.73-1.26)	0.774	0.85 (0.61-1.17)	0.311
Translocation partner	Others vs *MLLT3*	1.93 (1.32-2.81)	0.001	1.67 (1.08-2.60)	0.022	1.66 (1.24-2.22)	0.001	1.45 (1.04-2.04)	0.033
Protocol	Others vs AAML1031	1.11 (0.80-1.55)	0.531	1.22 (0.83-1.79)	0.307	1.06 (0.81-1.39)	0.665	1.19 (0.87-1.62)	0.285
pKMT2A7 group	G2 vs G1	2.32 (1.67-3.23)	<0.001	2.10 (1.43-3.11)	<0.001	2.41 (1.85-3.16)	<0.001	2.14 (1.55-2.94)	<0.001
HSCT at CR1	Yes vs No	1.08 (0.68-1.73)	0.735	0.86 (0.53-1.41)	0.549	0.57 (0.37-0.89)	0.014	0.45 (0.28-0.71)	<0.001

EFS = event-free survival; HR = hazard ratio; HSCT = hematopoietic stem cell transplantation; OS = overall survival; WBC = white blood cell count.

**Figure 5.**
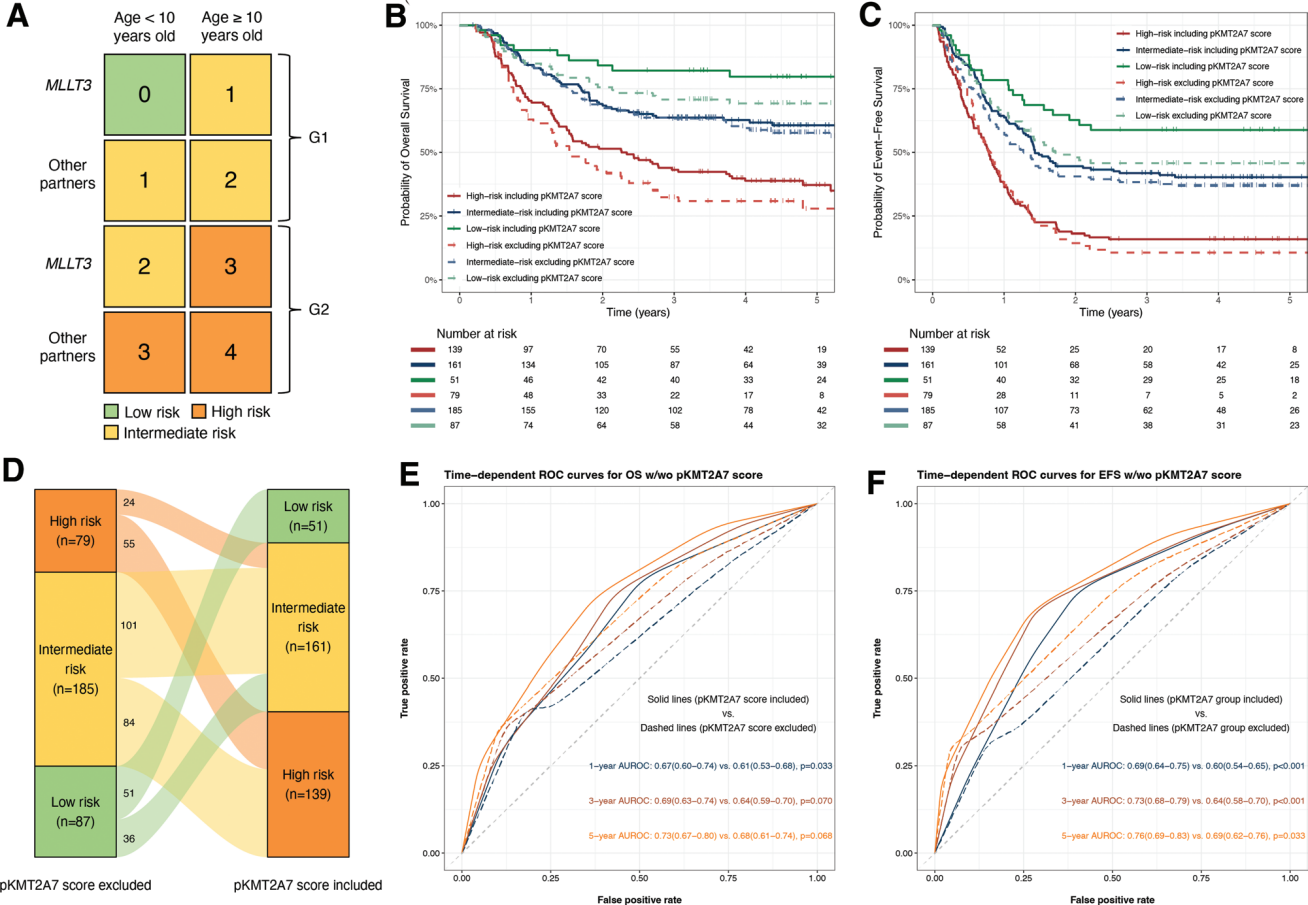
(A) The chart is presented to show the cumulative score of the prognostic system. In each cell of the chart, the score for a patient is calculated with the values of each predictor. Then, the cells of the chart are colored in accordance with the risk status: 0, low risk, 1–2, intermediate risk, and 3–4, high risk. (B,C) Kaplan-Meier curves for OS and EFS rates of risk groups stratified by the proposed prognostic system including (solid lines) and excluding pKMT2A7 score (dashed lines). (D) Different risk stratification including and excluding pKMT2A7 score. (E,F) Time-dependent ROC curves for OS and EFS comparing prognostic models including and excluding pKMT2A7 score. EFS = event-free survival; OS = overall survival; ROC = receiver operating characteristic.

To further refine the risk stratification, we incorporated independent risk factors for EFS into the prognostic model except for HSCT because it was a treatment rather than a clinical feature. Integer weights were assigned according to HRs in multivariable Cox regression of EFS. Factors with HRs of 1.0–2.0 were converted into a weight of 1.0, and HRs of >2.0 were converted into a weight of 2.0. The revised risk score was formulated as such: age at diagnosis (≥10 y old) × 1 + translocation partner (other than *MLLT3*) × 1 + pKMT2A7 score (G2) × 2 (Table 3). Using the new prognostic system, patients were stratified into 3 risk groups, low risk (0 score), intermediate risk (1–2 scores), and high risk (3–4 scores) (Figure 5A). The 5-year OS rates of low-risk, intermediate-risk, and high-risk groups were 79.8% ± 5.8%, 60.7% ± 4.0%, and 35.0% ± 4.6%, respectively (high risk versus low risk: HR, 4.29 [95% CI, 2.23-8.27], *P* < 0.001; intermediate risk versus low risk: HR, 2.19 [95% CI, 1.12-4.28], *P* < 0.001). The 5-year EFS rates of low-risk, intermediate-risk, and high-risk groups were 58.8% ± 6.9%, 40.2% ± 4.0%, and 16.0% ± 3.1%, respectively (high risk versus low risk: HR, 3.48 [95% CI, 2.18-5.55], *P* < 0.001; intermediate risk versus low risk: HR, 2.67 [95% CI, 1.04-2.68], *P* < 0.001). Meanwhile, low-risk group had the highest MRD negativity rate after course 1 followed by intermediate-risk and high-risk group (93.9% [31/33], 89.0% [89/100], and 74.7% [68/91], *P* = 0.009); however, the difference was not statistically significant for MRD negativity rate after course 2 (100% [27/27], 95.2% [79/83] and 92.6% [63/68], *P* = 0.418).

**Table 3:** Weighted score of each factor in the prognostic system

Variable	Weighted score		
	0	1	2
Age at diagnosis	<10 y old	≥10 y old	
Translocation partner	*MLLT3*	Other translocation partners	
pKMT2A group	G1		G2

We further evaluated how the prognostic system perform without pKMT2A7 score. By the same strategy, we assigned integer weights to the independent risk factors according to HRs in multivariable Cox regression of EFS. Results of multivariable Cox regression excluding pKMT2A7 score were summarized in Suppl. Table S2. Age at diagnosis (≥10 y old) and translocation partners (other than *MLLT3*) were assigned to 2 scores and 1 score, respectively. We defined patients with 0 score as low risk, 1–2 scores as intermediate risk and 3 scores as high risk. The newly proposed risk stratification outperformed the one that did not contain pKMT2A7 score with better discriminative ability especially in identifying low-risk patients (Figure 5B–D) and higher AUROC (Figure 5E,F).

### Clinical significance of the newly proposed risk stratification

**Figure 6.**
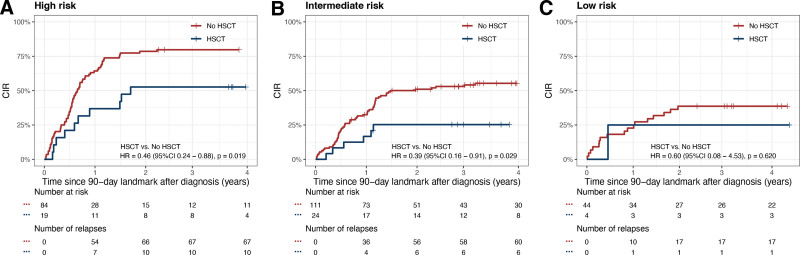
**CIR curves.** (A) high-risk, (B) intermediate-risk, and (C) low-risk groups stratified by newly proposed risk stratification. CIR = cumulative incidence of relapse.

After setting 90-day after diagnosis as landmark, there were 8.3% (4/48), 17.8% (24/135), and 18.4% (19/103) patients who underwent HSCT at CR1 in the low-risk, intermediate-risk, and high-risk groups, respectively. We examined whether HSCT at CR1 could improve the prognosis of each group by analyzing the CIR of each risk groups. HSCT significantly reduced CIR in both high- (HSCT versus No HSCT: 52.6% ± 12.0% versus 79.8% ± 4.5%, HR, 0.46 [95% CI, 0.24-0.88], *P* = 0.019, Figure 6A) and intermediate-risk group (HSCT versus No HSCT: 25.3% ± 9.2% versus 55.3% ± 4.9%, HR, 0.39 [95% CI, 0.16-0.91], *P* = 0.029, Figure 6B). The CIR was similar between patients who underwent HSCT and those who did not (HSCT versus No HSCT: 25.0% ± 25.0% versus 38.6% ± 7.5%, HR, 0.60 [95% CI, 0.08-4.53], *P* = 0.620, Figure 6C).

## DISCUSSION

Herein, we analyzed clinical and RNA-seq data of 351 pediatric *KMT2A*-r AML patients, and our findings suggested that transcriptomic features (pKMT2A7 score) can be informative of prognosis. Meanwhile, we identified some biological pathways that may be the underlying mechanisms causing the discrepancies of prognosis and provided foundation for further pathophysiology research. Finally, by incorporating clinical characteristics into pKMT2A7 score, we developed a new prognostic system and refined risk stratification for pediatric *KMT2A*-r AML, which outperformed risk stratification based on age and translocation partners and could be instructive in clinical decision-making.

In our study, the prognosis of pediatric *KMT2A*-r AML remained poor with 5-year EFS around 33% despite that over 95% patients were originally assigned as standard risk. This reflected the risk allocation of pediatric *KMT2A*-r AML needed urgent refinement. Recent years have seen many efforts made in developing transcriptome-based risk prediction, but they rarely focused on *KMT2A*-r AML. LSC17 score is a widely accepted model for risk prediction of AML, which was validated in pediatric cohorts as well. We proved that pKMT2A7 score had better discriminability than the LSC17 score, at least in the *KMT2A*-r AML. This could be attributed to several reasons: (1) The LSC17 score was originally developed on adult AML data, the discrepancies of transcriptome between pediatric and adult AML might result in reduction of predictive accuracy^24^; (2) Pediatric *KMT2A*-r AML is a highly heterogenous group, with some patients having favorable outcomes similar to core binding factor AML, whereas some carrying extremely poor prognosis. Consequently, *KMT2A* rearrangement often failed to be identified as a risk factor in the analysis including all kinds of pediatric AML.^21^ A model built on the entire AML cohort may lose some accuracy in discriminating patients in one specific subtype. Therefore, we recommend pediatric *KMT2A*-r AML be treated as a special entity with its own risk stratification.

It is very interesting that G1 group, the one with relatively better outcome, also had more patients under 10 years old and with *MLLT3* fusion partner, which were proved to be protective factors in multivariable analysis as well. This phenomenon raised the question that did pKMT2A7 score really confer prognostic information or it was confounded by other factors. However, pKMT2A7 score still remained the strongest predictor for survival when including all potential prognostic factors in multivariable analysis and subgroup analyses. Meanwhile, the fact that discriminability of the model reduced to a great deal when eliminating pKMT2A7 score further underscored its prognostic value. So, we think pKMT2A7 score reflected the heterogeneity within certain translocation partners. Still, larger cohorts and more integrated transcriptomic analyses are needed to address this problem.

Recently, a paper reported that apart from translocation partners, positivity of flow cytometry-based MRD at the end of induction 2 was also a strong predictor in pediatric *KMT2A*-r AML and suggested it being included into risk stratification.^22^ But our data showed that though there were only 5% patients who did not achieve MRD negativity after two courses, most patients still relapsed even with rapid MRD clearance, indicating risk stratification based on MRD at the end of course 2 might underestimate the risk for some patients. The biggest strength of our prognostic model is that we determine risk groups at very early stage without knowing treatment response, which can facilitate protocol design, for example, intensifying induction 2 or introducing more targeted therapy, to achieve a more profound remission and improve the prognosis of patients with higher risk.

HSCT is one of the major consolidation therapies for pediatric AML with adverse factors. Most current evidence supported that HSCT could improve the outcomes of pediatric *KMT2A*-r AML.^25,26^ Nonetheless, our findings indicated that only individuals categorized as high- and intermediate risk according to our prognostic system could potentially benefit from HSCT in CR1. Given the elevated therapy-related mortality associated with HSCT and its impact on long-term quality of life, the candidates and timepoint to receive HSCT should be carefully considered. Therefore, further refinement to the prognostic system is warranted to discover more heterogeneity and enhance the accuracy of HSCT candidate selection. Besides, our study also identified a small group (14.5%, 51/351) of patients with low risk, who shared similar outcomes with conventional low-risk AML such as AML with core binding factor fusion genes. For patients with low risk, HSCT at CR1 did not confer survival benefit, thus the improvement of prognosis relied on novel treatment approaches in addition to conventional chemotherapy.

Menin is one of the most important parts of the *KMT2A* complex and is critical to develop and maintain leukemogenesis through epigenetic modulation.^27,28^ The first menin inhibitor was developed in 2012 with the ability to down-regulate the target genes involving in oncogenesis and induce both apoptosis and differentiation of leukemia cells harboring *KMT2A* translocations. Subsequently, multiple preclinical studies demonstrated potent anti-tumor activity of menin inhibitor in vivo, paving the way for the ongoing clinical trial.^29,30^ Besides, menin inhibitor in combination with BCL2 inhibitors also exerted synergistic lethality in cell lines.^31^ These potent agents are promising to further enhance treatment effectiveness and reduce toxicity, especially for those low-risk and intermediate-risk patients in our prognostic model. In addition, we identified some biological pathways that might be associated inferior outcomes. Both dysregulation of cell cycle and metabolic abnormalities had abundant laboratory evidence showing their relevance with the pathogenesis of AML.^32,33^ Preclinical studies showed agents blocking cell cycle progression pathways was effective in leukemia cell lines with *KMT2A* translocations.^34^ Blocking these pathways may have a pivotal role in enhancing outcomes of *KMT2A*-r AML and possibly exempt some from undergoing HSCT.

There are some limitations of our study. First, our model was developed and validated based on limited cohorts, larger sample size is needed to validate our conclusions. Second, there were randomizations in the protocols used in this study, bortezomib in AAML1031 and gemtuzumab-ozogamicin in AAML0531, which could potentially bias the results. Third, due to the extensive range of translocation partners of *KMT2A*, we aggregated all others except for *MLLT3*, which could potentially under- or overestimate the risk of some translocation partners.

## AUTHOR CONTRIBUTIONS

JL, SZ, YCZ, and XZ designed the study. JL, SZ, and YW collected the data. JL and SZ analyzed the data and drafted the manuscript. PW, YCZ, and XZ made critical revisions to the manuscript for important intellectual content. YW, MR, LZ, WY, XC, YZ, YC, and YG provided guidance on statistical analysis and presentation of results. All authors approved the final manuscript and agreed to submit for publication.

## DATA AVAILABILITY

This study does not contain any research involving any human participants or animals performed by any of the authors. All datasets analyzed in this study are available at https://ocg.cancer.gov/programs/target/data-matrix. The core code used in this study is available at https://github.com/pediatric-ihcams/pKMT2A7-score.git.

## DISCLOSURES

The authors declare no conflict of interest.

## SOURCES OF FUNDING

This work was supported by the Ministry of Science and Technology of China (2019YFA0110803), the CAMS Innovation Fund for Medical Sciences (2022-I2M-1-022), the National Natural Science Foundation of China (82270144, 81870131), and the National Key R&D Program of China (2021YFE0106900).

## Supplementary Material


