# Supplementary figures and images for: Integration of Transcriptomic Features to Improve Prognosis Prediction of Pediatric Acute Myeloid Leukemia With KMT2A Rearrangement
Source: Hemasphere. 2023 Nov 22;7(12):e979. doi: 10.1097/HS9.0000000000000979 (PMC10666994; doi:10.1097/HS9.0000000000000979)

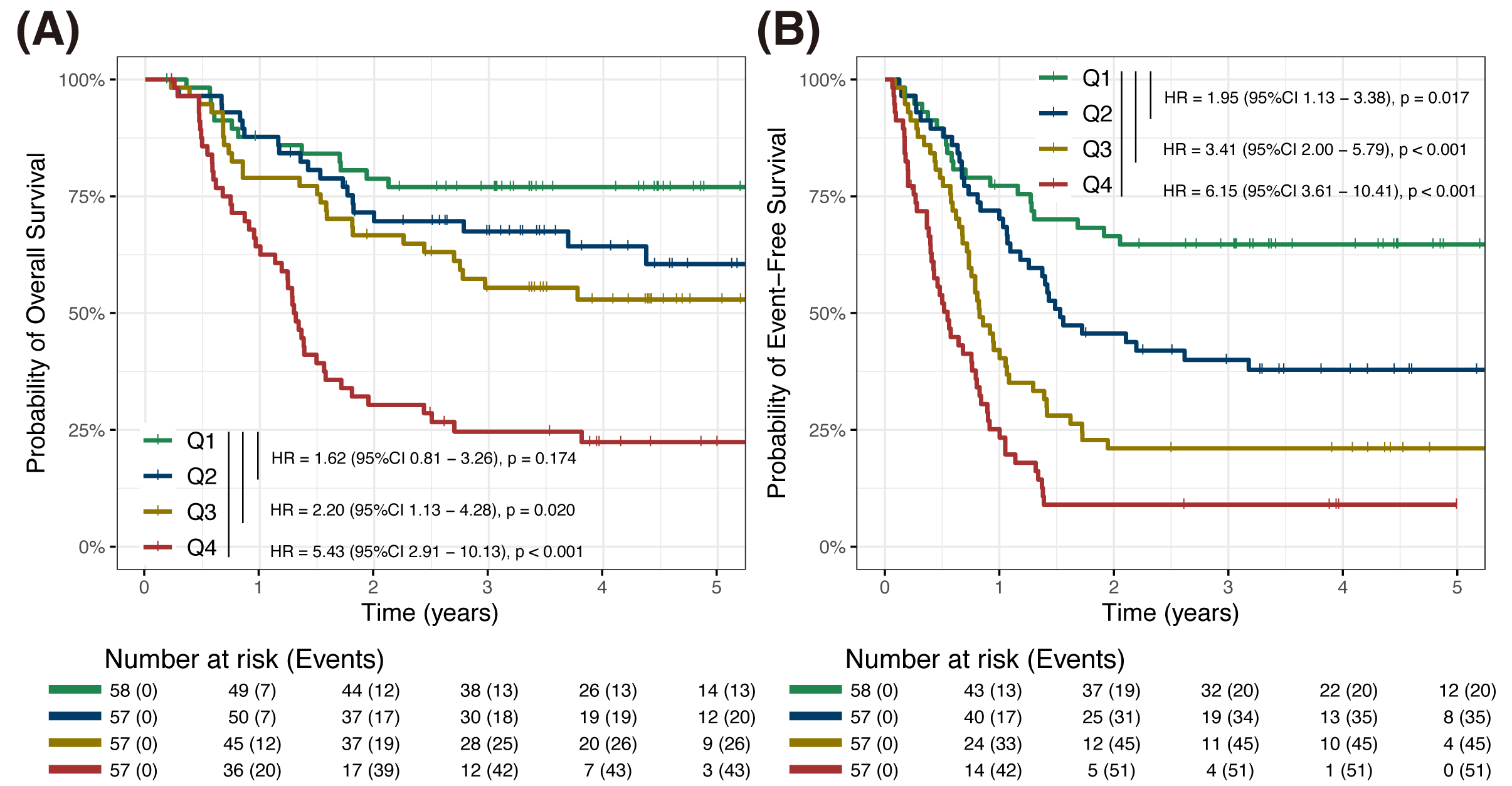

Supplement: Supplementary file 1 [file hs9-7-e979-s001.tif]

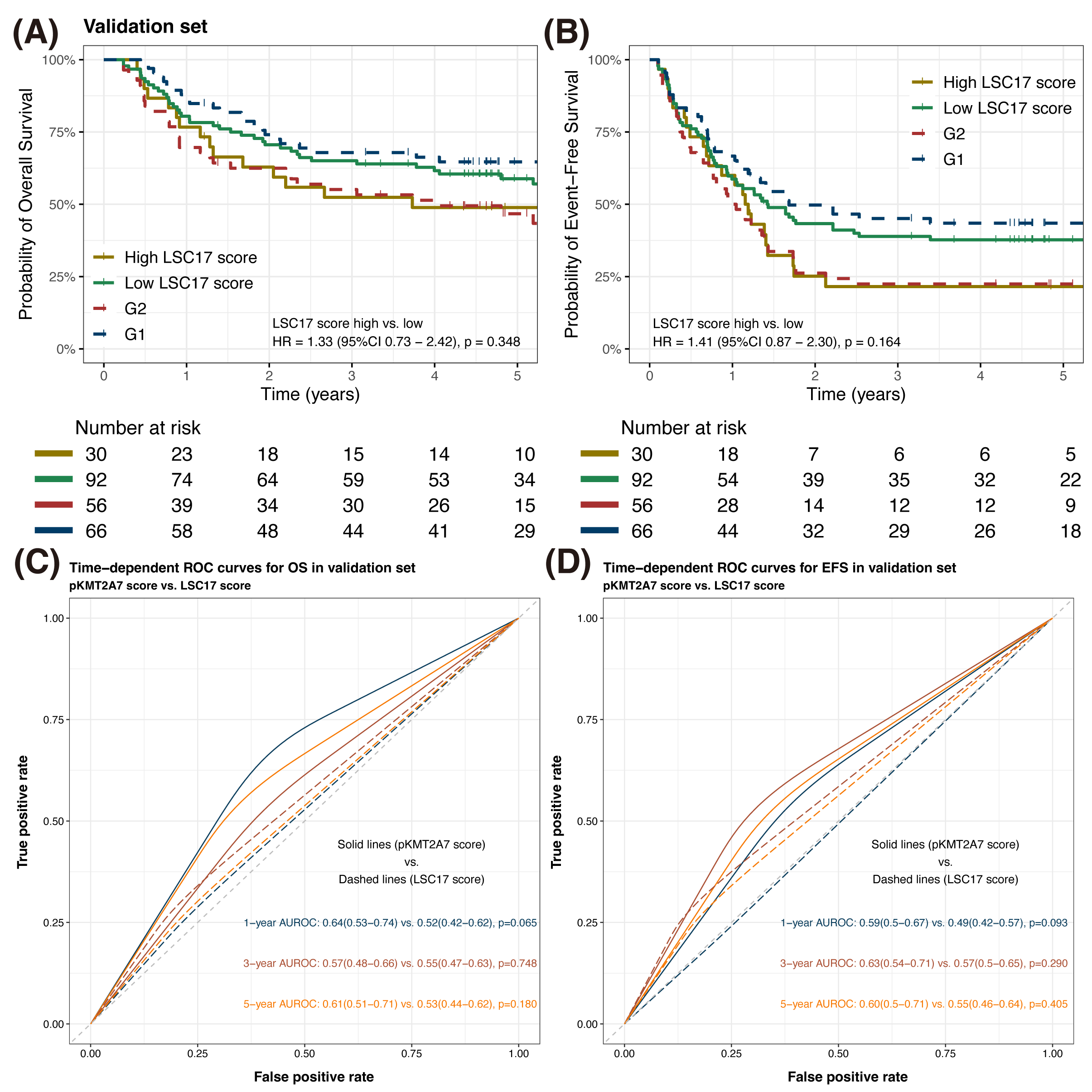

Supplement: Supplementary file 3 [file hs9-7-e979-s003.tif]

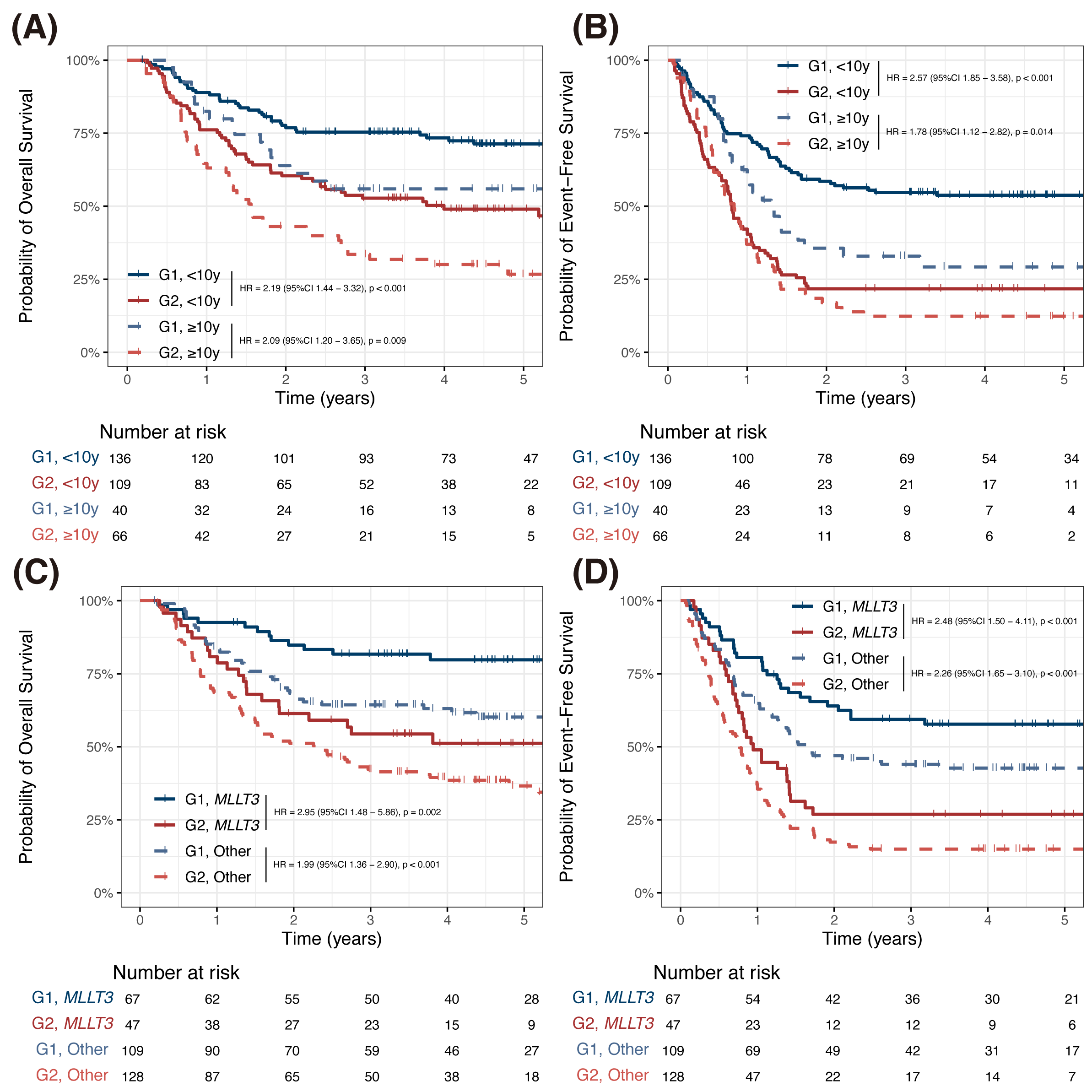

Supplement: Supplementary file 4 [file hs9-7-e979-s004.tif]
