# Supplementary material for: Integration of Transcriptomic Features to Improve Prognosis Prediction of Pediatric Acute Myeloid Leukemia With KMT2A Rearrangement
Source: Hemasphere. 2023 Nov 22;7(12):e979. doi: 10.1097/HS9.0000000000000979 (PMC10666994; doi:10.1097/HS9.0000000000000979)
Supplement: Supplementary file 2 [file hs9-7-e979-s002.docx]

| Variable | level | G1 (n=110) | G2 (n=119) | p |
| --- | --- | --- | --- | --- |
| Gender (%) | Female | 55 (50.0) | 55 (50.0) | 0.187 |
|  | Male | 55 (50.0) | 70 (58.8) |  |
| Age at diagnosis (years, %) | <10 | 87 (79.1) | 77 (64.7) | 0.019 |
|  | ≥10 | 23 (20.9) | 42 (35.3) |  |
| WBC at diagnosis (×10^9^/L, %) | <50 | 74 (67.3) | 72 (60.5) | 0.336 |
|  | ≥50 | 36 (32.7) | 47 (39.5) |  |
| Bone marrow blast (%, %) | <80 | 49 (44.5) | 50 (42.0) | 0.546 |
|  | ≥80 | 50 (45.5) | 51 (42.9) |  |
|  | NA | 11 (10.0) | 18 (15.1) |  |
| Translocation partner (%) | *MLLT3* | 44 (40.0) | 32 (26.9) | 0.049 |
|  | Other partner genes | 66 (60.0) | 87 (73.1) |  |
| *FLT3*-ITD (%) | No | 107 (97.3) | 115 (96.6) | 1.000 |
|  | Yes | 3 (2.7) | 4 (3.4) |  |
| CR status at end of course1 (%) | CR | 88 (80.0) | 92 (77.3) | 0.807 |
|  | not in CR | 20 (18.2) | 23 (19.3) |  |
|  | unevaluable | 2 (1.8) | 4 (3.4) |  |
| HSCT in 1st CR (%) | No | 92 (83.6) | 84 (70.6) | 0.058 |
|  | Yes | 11 (10.0) | 19 (16.0) |  |
|  | Unknown | 7 (6.4) | 16 (13.4) |  |

Supplementary Table 1. Clinical characteristic of G1 and G2 groups of training set.

WBC: white blood cell count; *FLT3*-ITD: FMS-like tyrosine kinase 3-internal tandem duplication; CR: complete remission; HSCT: hematopoietic stem cell transplant; NA: not available.
