# Supplementary material for: Integration of Transcriptomic Features to Improve Prognosis Prediction of Pediatric Acute Myeloid Leukemia With KMT2A Rearrangement
Source: Hemasphere. 2023 Nov 22;7(12):e979. doi: 10.1097/HS9.0000000000000979 (PMC10666994; doi:10.1097/HS9.0000000000000979)
Supplement: Supplementary file 5 [file hs9-7-e979-s005.docx]

|  |  | OS | | | | EFS | | | |
| --- | --- | --- | --- | --- | --- | --- | --- | --- | --- |
| Variables | Groups | Univariable analysis | | Multivariable analysis | | Univariable analysis | | Multivariable analysis | |
|  |  | HR (95%CI) | p | HR (95%CI) | p | HR (95%CI) | p | HR (95%CI) | p |
| Gender | Male vs. Female | 1.15 (0.84 - 1.57) | 0.399 | 0.94 (0.65 - 1.35) | 0.726 | 1.21 (0.93 - 1.57) | 0.153 | 1.00 (0.74 - 1.36) | 0.980 |
| Age at diagnosis (years) | <10 vs. ≥10 | 1.93 (1.4 - 2.66) | <0.001 | 1.94 (1.32 - 2.87) | 0.001 | 1.56 (1.19 - 2.04) | 0.001 | 2.00 (1.43 - 2.81) | <0.001 |
| WBC at diagnosis (×10^9^/L) | <50 vs. ≥50 | 1.23 (0.9 - 1.69) | 0.195 | 1.40 (0.97 - 2.04) | 0.073 | 1.22 (0.94 - 1.58) | 0.131 | 1.20 (0.88 - 1.62) | 0.250 |
| Bone marrow blast (%) | <80 vs. ≥80 | 0.91 (0.65 - 1.26) | 0.568 | 0.82 (0.56 - 1.20) | 0.299 | 0.96 (0.73 - 1.26) | 0.774 | 0.82 (0.59 - 1.13) | 0.228 |
| Protocol | Others vs. AAML1031 | 1.11 (0.8 - 1.55) | 0.531 | 1.26 (0.86 - 1.84) | 0.236 | 1.06 (0.81 - 1.39) | 0.665 | 1.14 (0.84 - 1.55) | 0.410 |
| Translocation partner | Others vs. *MLLT3* | 1.93 (1.32 - 2.81) | 0.001 | 1.89 (1.22 - 2.92) | 0.004 | 1.66 (1.24 - 2.22) | 0.001 | 1.64 (1.17 - 2.30) | 0.004 |
| HSCT at CR1 | Yes vs. No | 1.08 (0.68 – 1.73) | 0.735 | 0.87 (0.53 - 1.42) | 0.575 | 0.57 (0.37 – 0.89) | 0.014 | 0.48 (0.30 - 0.76) | 0.002 |

Supplementary Table 2. Uni- and multivariable Cox analysis of variables impacting OS and EFS excluding pKMT2A7 score.

WBC: white blood cell count; OS: overall survival; EFS: event-free survival; HR: hazard ratio; HSCT: hematopoietic stem cell transplantation.
